# Supplementary figures and images for: Embodiment of an artificial limb in mice
Source: PLoS Biol. 2025 Jun 5;23(6):e3003186. doi: 10.1371/journal.pbio.3003186 (PMC12140191; doi:10.1371/journal.pbio.3003186)

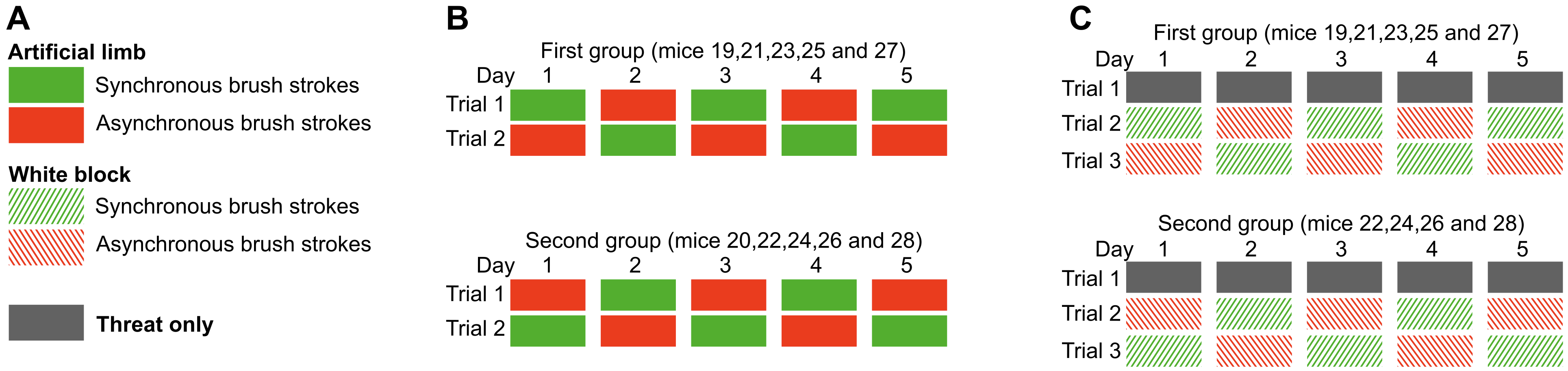

Supplement: S1 Fig — (A) Listing of all experimental conditions. (B) Order of the experimental conditions during the first experiment, in which ten EMX-Cre C57/BL6 mice received both synchronous and asynchronous stimulations on the artificial limb. (C) Second experiment, in which nine of the ten mice from B were tested on three additional, control protocols, 40 days after the first experiment. (TIFF) [file pbio.3003186.s001.tiff]

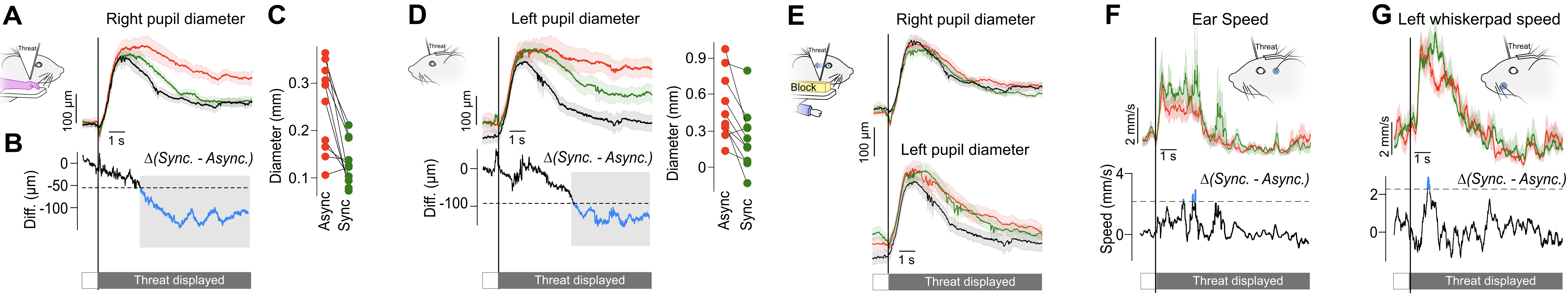

Supplement: S2 Fig — (A) Pupils are less dilated in response to the threat in the synchronous condition (green line) than in the asynchronous condition (red line). Average vertical diameter of the right pupil after the threat in the artificial limb condition, normalized relative to the mean position in the second before the threat (n = 10). Light background: SEM. (B) Average difference between the right pupil diameter between the two conditions in A. Blue sections: significant differences (Bootstrap based test p < 0.05). Black dashed line: significance threshold. Light gray background: time window selected for further investigation. (C) Average difference in the time window identified in B, for each individual mouse, in the synchronous versus asynchronous condition. (D) Difference between the sync/async contrasts observed in the artificial limb versus Block conditions. Blue sections: significant differences (Bootstrap based test p < 0.05). Black dashed line: significance threshold. (E) Same as A–D for the left pupil diameter. (F) Same as A, B, D for the absolute value of the instantaneous speed of the left ear. The average left ear speed following a threat increased more in synchronous versus asynchronous pairings with the artificial limb. This difference crossed at several time points the significance threshold. We did not find a significant ear movement difference between the threat response in the conditions of Artificial limb versus Block. (G) Same as F for the speed of either the B1 or C1 left whisker. The data and code underlying this figure is available in the following repository: https://doi.org/10.5281/zenodo.14635566. (TIFF) [file pbio.3003186.s002.tiff]
