# Supplementary material for: Embodiment of an artificial limb in mice
Source: PLoS Biol. 2025 Jun 5;23(6):e3003186. doi: 10.1371/journal.pbio.3003186 (PMC12140191; doi:10.1371/journal.pbio.3003186)
Supplement: Attachment — Submitted filename: response_to_reviewers.docx [file pbio.3003186.s005.docx]

Dear Dr. Schnell,

We thank the reviewers and editors for their kind interest in our work. We have now addressed the points raised by the reviewers, and taken this opportunity to improve the manuscript.

Below, we have addressed all reviewer comments.

REVIEWS:

Reviewer #1:

Dear Editors and authors,
In a new study Hayatou et al. aimed to establish a rodent model of the widely used "rubber hand illusion" paradigm in humans. This is an important task. While previous studies using the human paradigm allowed studying brain activity correlated to embodiment of artificial objects and thereby provided insight into the brain-wide organization, the neural- and circuit mechanisms underlying embodiment could not be investigated so far. This is mainly due to a lack of a laboratory animal model. The authors therefore elegantly adapted the human paradigm to rodents by using certain facial expression parameters as a readout for embodiment. In particular, they show that mice adjust their eyes gazing at the moving threat stimulus and that this adjustment is most pronounced if mice were previously brushed synchronously at their real and their artificial forepaw. If the brushing was performed non-synchronously the authors observed less gaze adjustments and if no brushing was preceded the threat stimulus the gaze adjustment was almost absent. The authors also performed proper control experiments. E.g. they showed that an object, which did not resemble details of the real forepaw generated less gaze adjustments in response to a threat stimulus, suggesting that that object was not embodied as strongly as the more realistic artificial limb.

Taken together, this study provides an important methodological advancement, which may be used in the future to probe brain functions related to body coordination and perception and possibly to address mechanistic questions regarding clinical conditions such as phantom limb pain. I strongly support publication of this work in PLOS Biology, however, the authors need to address some minor issues that may improve the quality of the manuscript.

We thank the reviewer for this very supportive review. Below we address all the points that were raised.

Minor: 
-At the end of the introduction the authors state: "Consistent with the human rubber hand illusion, the mice focused significantly more…". Here the authors should provide citations that show this gaze effect in humans.

Thank you for raising this point. Although some studies investigated gaze behavior during the brushing itself, and have found some correlation between fixation count and embodiment (Krogmeier & Mousas, 2020), to the best of our knowledge no study has quantified the gaze at the time of the threat being applied to the rubber hand. Nonetheless, while direct evidence is missing, previous research consistently demonstrates stronger subjective and physiological responses under synchronous conditions (Armel & Ramachandran, 2003; Botvinick & Cohen, 1998; Ehrsson et al., 2007) which is consistent with our data.

To clarify this point, we have therefore revised the sentence as followed: *“**Consistent with the broader literature on the rubber hand illusion* (Armel & Ramachandran, 2003; Ehrsson et al., 2007)*, we observe a stronger behavioural response under synchronous conditions. In our case, this is reflected by a significant gaze shift toward the incoming …”*

-The authors show 3 different experiments all carried out with the same mice. While this approach has the advantage that one gets within-animal data it has the disadvantage that possible habituation processes cannot be excluded. The authors should address this in the discussion.

We thank the reviewer for pointing this lack of discussion in the initial manuscript. Here is the paragraph we added to the section “The challenge of response variability”:

*Another source of variability is the potential habituation of the mice to the threat that we used to probe the illusion strength (Ornitz & Guthrie, 1989; Riemer et al., 2015). To minimize this issue, we presented the limb threat to the mice only twice a day during 5 days (synchronous and asynchronous conditions). We then waited 40 days before presenting a new set of conditions to the same mice (Supplementary Figure 1). Despite this limited exposition to the forelimb threat, we expect that part of the smaller response to the Block experiment (Figure 3) may be attributable to this threat habituation, although it is unlikely that it could be key to the significantly smaller synchronous/asynchronous difference in gaze shift in the Block experiment when compared to the Limb experiment.*

-In Figure 4 it does not become evident why in panel F (top) there is no depiction of analogous traces shown in panel A (top, green, red and black traces). The authors should have this data, so why is it not shown?

Our rationale was that this was a repeat of data shown in panels D,E of Figure 4 (no pairing) and data shown in Figure 3 (block pairing). Given the interest of the reviewer in seeing a complete picture, we have now added the raw data in F. The updated figure is shown below and has been integrated in the manuscript.

-In the discussion the authors provide valid arguments why the head and the forepaw were fixed. It may, however, still make sense to analyze the restrained forepaw/limb and see whether certain twitches (potentially of the fingers?) may also correlate with the degree of embodiment. Also, one may think of training mice to leave their forepaw at a desired place, which would make the paradigm more complicated but also more similar to the human paradigm. It would be good if the authors discuss this issue.

As suggested by the reviewer, we did look at the restrained forelimb movements in the hope of residual movements that would be revealing of embodiment in the same way as the pupil. However, similar to several other body features that we tracked, we failed to find meaningful embodiment-related forelimb movements.

Also, at the start of our project, we spent several months training mice to hold their forelimb steady, with limited success: the resulting forelimb position was too variable and too transient to enable successful brush stroke stimulations.

In the discussion section entitled “Towards the tracking of facial mimics for embodiment”, we added the following text:

*“**Using the high-speed imaging of a large part of the mouse body during the embodiment experiment, we could also search for signs of embodiment beyond the mouse face. In particular, we asked if the hand-cuffed forelimb displayed retraction movements in reaction to the threat. However, the paw being securely restricted, the attempted movements may not have been visible enough to be captured. Overall, we found that embodiment-related movements could only be detected on the face of the mice.”*

And in the section “Position of the artificial limb with respect to the physiological limb”, we added the following text:

*Note that instead of handcuffing, we initially attempted to condition the mice to hold their forelimb in place, and to accept brush strokes on the forelimb without retracting it. However, we never achieved the repeatability and time stability of the forelimb posture that was required to induce the embodiment then probe it.*

-The authors also discuss the non-intuitive results of their supplementary figure 2. There they show that pupil diameter was bigger in the asynchronous condition compared with the synchronous one. The authors state that cognitive processes may be involved in pupil diameter control but how this explains the observed results remains elusive. As far as I understand these results are difficult to match with existing findings from human studies as the degree of embodiment is usually positively correlated with levels of arousal and not negatively (as suggested by the results in supplementary figure 2). This issue should be discussed more intensively.

We thank the reviewer for raising this and leading us to think more about these results. We have concluded that another and more straightforward explanation might be the difference of luminance that might be experienced by the mice across the two conditions, as they either looked strongly in the direction of the bright white menace (synchronous condition) or not (asynchronous). We have added the following sentences to the discussion paragraph entitled “Contributions to changes in pupil diameter”:

*This difference in pupil diameter did not match our expectations, as we had initially hypothesized that the incoming threat should trigger a stronger emotional reaction in the synchronous than in the asynchronous condition, and therefore produce a larger pupillary dilation.*

*There are several confounding factors that may explain this pupillary behavior. First, in the synchronous condition, the mice focused their gaze towards the bright white plastic threat more than in the asynchronous condition. They may therefore have been exposed to a higher level of illumination, consequently reducing their pupillary diameter. Second,…*

-In the methods the authors should state at which angle the arrowhead-like white plastic was moved (in horizontal and vertical dimension).

The menace was attached to a rod that was rotated 180° by a servomotor, from a hidden position to the threat position. We have added this information to the method section “Design of the artificial paw illusion”.

Reviewer #2: This study describes what could become a useful resource for trying to understand circuitry underlying limb embodiment and how it operates in the mammalian brain. Namely, it introduces an assay which replicates in mice similar behaviors to those found in the 'rubber hand illusion' (RHI) in humans. 

My sense of this manuscript is that it is more a novel resource than a report of novel findings and insights. It seems to me that the value provided by replicating the RHI in mice would be to allow investigation of its neural circuit basis in a tractable model, which the manuscript lays the ground for, while stopping short of providing any neural evidence. 

More specifically: as the authors say in the Discussion, there has been controversy over whether human RHI arises purely from bottom-up multimodal integration leading to embodiment, or whether it is affected by top-down phenomena such as expectation and hypnotizability (Lush et al, 2020; Thériault et al, 2022). The current manuscript provides evidence suggesting that something similar to the human phenomenon is generated in mice and probably emerges without the need for hypnotizability, therefore providing a useful resource/experimental model to the community. This might lead to mechanistic accounts of RHI emergence through multimodal integration in mice, which could be highly interesting and relevant. This manuscript does set up an appropriate behavioral design but I don't think its novel findings are substantive enough for publication as a PLOS Biology research article. 

We thank the reviewer for their interest in the contribution of our work to the ongoing debates regarding neural mechanisms of the rubber hand illusion in humans, and its usefulness as a platform for further studies on the mechanisms using the mouse model.

Are data values properly aligned to axes in all figure panels? In Fig. 3B, the green and red curves seem to start at about the same level, and the green curve then stays above the red curve. Yet in Fig. 3C the difference value starts out highly negative, and then becomes negative again about a second after the end of W2, when green is still clearly greater than red in panel 3B. Please check alignment to axis values of these and other plots. 

We agree that reconciling these curves could appear quite puzzling to the reader, as detailed by the reviewer. This apparent discrepancy is due to a display and analysis choice we made. Through the manuscript, the raw data is shown with the average of the 120 s baseline subtracted (for instance in Figure 3B, see methods) in order to show clearly the amplitude of the threat response from the baseline. In contrast, during the corresponding significance analysis (Figure 3C), we did not subtract the baseline to compute the bootstrap distribution, reasoning that it could bias the detection of significant differences between the synchronous and asynchronous conditions. This explains that baseline effects are included in the outcome of the significance analysis, for instance in Figure 3C, but not visible in the individual curves display, for instance in Figure 3B.

We have explained it in an additional paragraph of the method section “Statistical analysis”:

*Note that in the significance analysis, there is no subtraction of the baseline, leading to non-zero synchronous/asynchronous difference at the start of the threat (for instance in Figure 3C) while the baseline is subtracted for the raw pupil positions (for instance in Figure 3B), in order to show the threat response amplitude.*

Reviewer #3: This paper is an elegant and novel contribution to the literature. The authors perform ingenious, well designed experiments to develop a paradigm for the study of limb embodiment in mice. With the current arsenal of tools for dissecting neural circuitry in this model, this could represent a panoply of new questions.

This reviewer could not find fault with the science; the only nagging big picture question was "how much does our inability to communicate with mice limit what can be done here?"  The authors address this in the discussion, especially from the perspective of what can be correlated with various cognitive and physiological states (eye movements, whisking). So it's more of a curiousity that criticism.

There were a couple tiny errors, otherwise the manuscript is well written and presented and publishable.

We are grateful for the reviewer strong interest for our work. We certainly believe that improving our ethological understanding of the mice communication, in particular through facial expression, will be key here.

We have corrected the minor issues pointed out below.

INTRO:

This sentence not grammatical:

Further, the lack of embodiment of prosthetic substitutes participates in the sensations that are perceived as arising from the "phantom" of the missing limb, including painful perceptions4.

We have corrected this sentence to convey its intended meaning:

*In contrast, prosthesis embodiment is associated with a reduction in the sensations arising from the “phantom” of the missing limb, including painful perceptions.*

discussion

typo - laugher -> laughter
Thank you for pointing it out.
